# Dietary, Lifestyle, and Children Health

**DOI:** 10.3390/nu15102242

**Published:** 2023-05-09

**Authors:** Xiaoran Yu, Zhiyong Zou

**Affiliations:** Institute of Child and Adolescent Health, School of Public Health, Peking University, Beijing 100191, China; yuxr0811@outlook.com

Childhood is a critical period for the development of a healthy lifestyle and the prevention of chronic diseases in adulthood. However, the prevalence of childhood obesity is increasing and unhealthy lifestyles are becoming an epidemic, posing a potential future burden of adult chronic disease. Therefore, in order to assess children’s health behaviors and to reduce the risk of chronic non-communicable diseases in early adulthood, this Special Issue of *Nutrients*, “Dietary, Lifestyle, and Children Health”, features 28 original studies and 3 systematic reviews on dietary or other lifestyle behavioral factors about children or adolescents. These findings may help to broaden our knowledge and to open up new research directions.

Dietary quality and diversity are as essential to human health as air is to human life. The study by Callum Regan [1] indicated that higher diet diversity and increased fruit and vegetable consumption could be a strategy to improve health-related quality of life among adolescents. Three papers in this Special Issue explored the relationship between vegetable and fruit intake, and childhood illness. These studies reported that moderate fruit consumption was associated with lower odds of lipid disorders [2] and that high fruit and vegetable intake (FVI) could reduce asthma-related illness [3]. However, insufficient FVI and low potassium intake aggravate early renal damage in children [4]. Cereals are a major source of dietary energy and a good source of many micronutrients and dietary fiber. The article by Xiaotong Wang and Tongtong He [5] found that high soy food intake (>3 times/week) was significantly associated with a lower prevalence of some chronic diseases in southern Chinese children. In addition to the child’s own diet behavior, maternal healthy lifestyle factors are associated with their offspring’s health. Dietary patterns during pregnancy were found by Siyuan Lv and Rui Qin to possibly have cumulative effects on an offspring’s neurodevelopment [6]. Regarding lactation, there is clear evidence of the importance of breastfeeding, compared with formula feeding, in the protection against chronic diseases. However, research by Jiajia Dang confirmed that prolonged breastfeeding (≥12 months) is detrimental and associated with an increased risk of offspring obesity [7]. 

Healthy diet patterns have a positive effect on the prevention of chronic non-communicable diseases in children. Given the malnutritional problems prevalent among children and adolescents, health education focusing on behavior intervention and nutrition education are necessary for containing nutritional problems among children [8]. An important prerequisite for better regulation of children’s diets is the monitoring and assessment of children’s dietary behavior. In this Special Issue, a systematic review written by Anne Krijger [9] explored many dietary and lifestyle screening tools, however, with poor prospects for the application of these screening tools. There is a need for an easy-to-administer screening tool for children in order to improve a child’s lifestyle at an early age. Additionally, in this Special Issue, Sheng Ma [10] first described the most commonly consumed food items, in terms of 29 food groups, nationally and by province and developed a low-burden, food group-based diet quality questionnaire (DQQ) for China to evaluate diet quality at the population level. Immediately afterward, Huan Wang et al. [11] confirmed that DQQ could be a valid tool to assess diet quality for Chinese children and adolescents. The Mediterranean diet is one of the dietary patterns with the healthiest outcomes, which has a positive role in protecting people from chronic non-communicable diseases. A longitudinal study found that children who adhere well to the Mediterranean diet have a lower risk of transient glomerular impairment [12]. Another randomized pilot trial indicated that a nutritional intervention based on the Mediterranean diet helps to reduce high glycated hemoglobin (HbA1c) and insulin levels and, therefore, to reduce the prevalence of prediabetes [13]. Their findings further underscore the necessity of early development of a healthy diet and contribute to providing some basis for establishing a targeted dietary intervention for children with certain diseases. 

In addition to healthy dietary patterns, other lifestyle factors, including a healthy body mass index, regular exercise, no smoking, and sufficient sleep duration, are associated with a lower incidence of chronic non-communicable diseases and longer life expectancy. Two papers in this Special Issue focus on the impact of sedentary behavior and digital screen time on chronic disease risk. First, the study by Ning Yin [14] indicated that higher sedentary behavior time is associated with a higher risk of metabolic syndrome components among children aged 6–14. Moreover, Agata Rockad et al. [15] surveyed the parents of preschool and school-aged children during the COVID-19 blockade in Poland and found that the majority of children were exposed to screens during meals, which is a risk factor of obesity. Additionally, it may negatively affect physical activity. This suggests an urgent need to develop effective strategies to limit excessive screen time and promote healthy eating habits and physical activity in children.

To summarize, the studies featured in this Special Issue are critical in identifying and assessing dietary factors, as well as other healthy lifestyle factors during childhood and adolescence. These findings not only will help to regulate eating behaviors and to improve the quality of children’s diet but also will help healthcare professionals to manage healthy behaviors in children, thereby reducing the prevalence of childhood chronic diseases.
